# ECG markers of malignant arrhythmias and in‐hospital outcome of COVID‐19 pneumonia

**DOI:** 10.1002/joa3.12506

**Published:** 2021-01-24

**Authors:** Foaad Shaghee, Hussein Nafakhi, Mohammed Alareedh, Ahmed Nafakhi, Karrar Al‐Buthabhak

**Affiliations:** ^1^ Internal Medicine Department Jabir ibn Hayyan Medical University Faculty of Medicine Kufa Iraq; ^2^ Internal Medicine Department Medicine College, university of Kufa Najaf Iraq; ^3^ Research Unit Najaf Health Bureau Ministry of Health Najaf Iraq

**Keywords:** COVID‐19, ECG, outcome, pneumonia, QTc

## Abstract

**Background:**

ECG abnormalities associated with COVID‐19 pneumonia and adverse outcome are undefined and poorly described in prior studies.

**Objectives:**

To assess the predictive role of baseline ECG markers of increased risk of malignant arrhythmias and cardiac death for adverse in‐hospital outcomes.

**Patients and methods:**

A retrospective study included 93 patients of newly diagnosed COVID‐19 with features consistent with pneumonia who were admitted to the hospital from August 20 to September 20, 2020. The main outcomes were defined as receiving mechanical ventilation, in‐hospital cardiac arrest, length of ICU stay, and degree of lung damage according to computed tomography (CT) score.

**Results:**

Increased QTc (QT corrected) interval, Tp‐e (T from peak to end) interval, and transmural dispersion of repolarization (TDR) were independent predictors of prolonged ICU stay (*P* < .0001) after adjustment for baseline clinical characteristics. Increasing age (*P* < .0001) followed by increased QTc interval (*P* = .02) and history of chronic lung disease (*P* = .04) were independent predictors of extensive lung damage. The independent predictors for in‐hospital cardiac arrest were increased QTc (*P* = .02) followed by increasing age (*P* = .04) and increased Tp‐e interval (*P* = .04).

**Conclusion:**

Repolarization abnormalities on baseline ECG may be useful prognostic markers in patients with COVID‐19 pneumonia.

## Graphical abstract

There is scant data as of yet that address the predictors of increased risk of adverse outcome of COVID‐19 pneumonia, particularly during acute phase of infection. Baseline ECG repolarization abnormalities may be useful prognostic markers in patients with COVID‐19 pneumonia according to the results of this study.

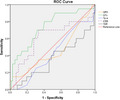

## 1 INTRODUCTION

Coronavirus disease 19 (COVID‐19) is caused by Severe Acute Respiratory Syndrome Coronavirus‐2 (SARS‐CoV‐2).^1^ The lung is the most common organ to be affected by COVID‐19 presenting as pneumonia and may progress to acute respiratory distress syndrome requiring hospitalization, which is the main cause of mortality in the acute phase of the disease.^2^, ^3^

Cardiac injury and ECG disturbances are not uncommon in COVID‐19 pneumonia and may lead to adverse short‐term outcomes. Cardiac complications such as QTc (QT corrected) interval prolongation, ventricular arrhythmia, or even sudden cardiac death are reported in patients with COVID‐19.^4^, ^5^

In the literature, ECG abnormalities and their association with clinical adverse outcomes related to COVID‐19 pneumonia are still undefined and poorly described in prior studies.^6^ The available data have suggested that ECG changes recorded during the early presentation or hospital stay can be used as a predictor for disease severity related to the clinical course of viral infection.^4^

Over the past decade, some electrocardiographic markers, including transmural dispersion of repolarization (TDR) of left ventricle measured by T peak to end interval (Tp‐e) and Tp‐e/QTc and index of cardiac electrophysiological balance (iCEB) measured by QTc/QRS, have emerged as novel ECG markers correlated with myocardial fibrosis, repolarization and conduction abnormalities, arrhythmic events, and even sudden cardiac death in various cardiovascular diseases.^7^, ^8^ Moreover, these ECG markers have suggested being a prognostic marker of adverse short‐ and long‐term outcomes in patients with coronary artery disease.^9^, ^10^

The main aim of the study was to assess the baseline ECG markers of repolarization and depolarization disturbances associated with increased risk of malignant arrhythmias and cardiac death, including QTc, Tp‐e, TDR, and iCEB, with short‐term clinical outcomes for patients with COVID‐19 pneumonia.

## 2 PATIENTS AND METHODS

This observational retrospective study included patients with newly diagnosed COVID‐19 pneumonia who were presented to the outpatient clinic and admitted to Al‐Sader teaching hospital in Al‐Najaf governorate from August 20 to September 20, 2020. All patients were presented with features consistent with COVID‐19 pneumonia based on clinical symptoms and radiological findings. Diagnosis of COVID‐19 infection was either by (1) positive nasopharyngeal swab by real‐time polymerase chain reaction (PCR) or (2) negative swab for COVID‐19 but the clinical symptoms suggestive of viral illness (cough, fever, and shortness of breath) plus radiological features of COVID‐19 infection on lung imaging.^3^ At hospital admission, the baseline clinical characteristics were recorded using medical records and collected by physicians at the study site‐level. The baseline clinical characteristics were: age, sex, hypertension, diabetes mellitus, chronic lung disease, smoking, body mass index (BMI), and previous coronary artery disease. The severity of lung damage by COVID‐19 pneumonia was assessed by computed tomography (CT) scan score according to a previously reported method for assessment of lung involvement and severity in COVID‐19 infection.^3^ The main outcomes for the study were defined as receiving mechanical ventilation, in‐hospital cardiac arrest, length of ICU stay, and degree of lung damage according to CT score. Approval of this study was provided by our medicine College Board and verbal consent was obtained from patients or relatives.

### 2.1 ECG examination

The 12‐lead ECGs were obtained for all patients within 24 hours of hospital admission with a paper speed of 25 mm/s and voltage of 10 mm/mV by using a standard ECG system (Marquette Electronics, WI, USA) while the patient was resting in the supine position. ECG markers were measured manually by two cardiologists blinded to the patient's status, using calipers and a magnifying glass. Any disagreement in ECG interpretations between cardiologists was resolved by consensus. QRS duration in milliseconds (ms) was measured from the initiation of the Q or R waves until the end of the R or S waves. Tp‐e interval in ms was measured from the peak of the T wave to the end of the T wave in the precordial leads. The mean value of the measurements was used in the analysis. The QT interval in ms was measured from the beginning of the QRS complex to the end of the T wave. Measured QT intervals were corrected by Bazett's formula (QT/(RR interval)1/2) and defined as corrected QT interval (QTc). The Tp‐e/QTc ratio and QTc/QRS were calculated from these measurements.^7^, ^8^

### 2.2 Statistical analysis

Statistical analysis was performed using SPSS ver. 23.0 (SPSS Inc, Chicago, IL, USA). *P*‐value of < .05 was chosen for statistical significance. Baseline clinical data of the patients and ECG markers were expressed as mean ± standard deviation for continuous variables or as numbers with percentages for categorical data. Receiver operating characteristic (ROC) curve analysis was used to assess the most optimal cut‐off values of ECG markers as predictors for in‐hospital cardiac arrest. These results were reported as area under the curve (AUC) with sensitivity and specificity. Univariate and multivariate logistic regression analyses were used to calculate the odds ratio and confidence intervals [OR (CI)], and assess the association of ECG markers and baseline characteristics with short‐term outcomes, including receiving mechanical ventilation, in‐hospital cardiac arrest, length of ICU stay, and degree of lung damage according to CT score. Baseline clinical characteristics, including age, sex, hypertension, diabetes mellitus, chronic lung disease, smoking, body mass index (BMI), and previous coronary artery disease, and all ECG markers underwent univariable logistic regression to the clinical outcome. Those with a *P* value of < .05 were candidates for inclusion in the final multivariable logistic regression analysis.

## 3 RESULTS

The initial study cohort included 118 patients with COVID‐19 pneumonia and followed up for short‐term outcomes during hospital admission until discharge or death. Of whom, 25 patients were excluded because of discharge on their responsibility before completion of treatment (n = 19), refuse to receive intubation (n = 3), or un‐interpretable ECG (n = 3), leaving 93 patients for final analysis.

Of the 93 patients enrolled in the study, 65 (70%) patients had mild‐moderate COVID‐19 pneumonia and 28 (30%) patients had severe COVID‐19 pneumonia according to CT scan score of lung damage and accompanying systemic and respiratory clinical features. Nineteen (20%) patients had in‐hospital cardiac arrest, and 23 (25%) patients received mechanical ventilation. The mean ICU stay length was 13 ± 2 days. The most common baseline comorbidities among 93 patients were age >50 year (66%), obesity (54%), hypertension (39%), diabetes mellitus (29%), previous coronary artery disease (16%), chronic lung disease (15%), and smoking (4%). The baseline characteristics and ECG marker values are summarized in Table 1.

**TABLE 1:** Patients' characteristics

Clinical characteristics	n (%) or mean ± SD
Age (years)	53 ± 14
Age > 50 year	62 (66%)
Male	41 (44%)
BMI	30 ± 5
Obesity	50 (54%)
Hypertension	36 (39%)
Diabetes mellitus	27 (29%)
Smoking	4 (4%)
Chronic lung disease	14 (15%)
Previous coronary artery disease	15 (16%)
ECG markers	
QRS duration, ms	90 ± 13
QTc interval, ms	430 ± 34
Tp‐e interval, ms	70 ± 12
iCEB (QTc/QRS)	4.8 ± 7
TDR (Tp‐e/QTc)	0.16 ± 0.0
Outcomes	
Length of ICU stay (days)	12 ± 8
ICU admission (n%)	31 (33%)
Degree of lung injury	39 ± 25
Cardiac arrest (death)	19 (20%)
Mechanical ventilation use	23 (25%)

Abbreviations: BMI, body mass index; iCEB, index of cardiac electrophysiological balance; ICU, intensive care unit; ms, milliseconds; SD, standard deviation; TDR, transmural dispersion of repolarization; Tp‐e, T from peak to end interval.

**FIGURE 1:**
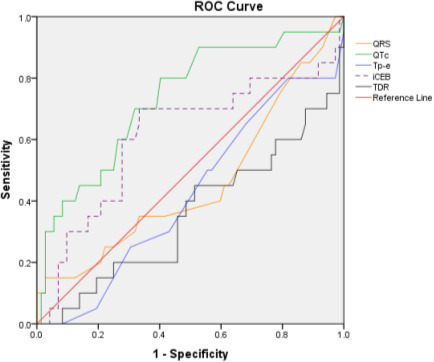
Receiver operating characteristic (ROC) curve for predicting in‐hospital death. QTc having the highest area under ROC (0.733) with most optimal cut‐off value of 435 ms for predicting in‐hospital death

The area under ROC curve for predicting in‐hospital death was 0.733 for QTc, 0.458 for QRS, 0.422 for Tp‐e, 0.611 for iCEB, and 0.356 for TDR. The most optimal cut‐off values of ECG markers for predicting in‐hospital cardiac arrest assessed by ROC curve analysis were: QTc = 435 ms (70% sensitivity and 32% specificity), QRS = 85 ms (70% sensitivity and 32% specificity), Tp‐e = 57 ms (80% sensitivity and 83% specificity), TDR = 0.13 (60% sensitivity and 79% specificity), and iCEB = 4.9 (70% sensitivity and 33% specificity) (Figure 1).

### 3.1 Univariate analysis

Increased QTc interval, Tp‐e interval, and TDR were significantly associated with increased risk for prolonged ICU stay, extensive degree of lung damage, and in‐hospital cardiac arrest (OR (CI) 0.6 (0.1‐0.7), *P* < .0001, OR (CI) 2 (0.9‐4), *P* < .0001, and OR (CI) 4 (1‐5), *P* < .0001, respectively), while iCEB showed no significant increased risk for adverse outcomes. On the other hand, no significant association was observed between QTc interval, Tp‐e interval, TDR, and iCEB with the use of mechanical ventilation (Table 2).

**TABLE 2:** Univariate analysis of baseline ECG markers and clinical outcomes

ECG markers	ICU length stay		Lung injury		Death		Mechanical ventilation	
	OR(CI)	*P*	OR(CI)	*P*	OR(CI)	*P*	OR(CI)	*P*
QRS	0.4(−0.2‐0.7	.22	0.4(−1‐2)	.37	1(0.9‐1)	.43	1(0.9‐1)	.31
QTc	0.6(0.1‐0.7)	<.0001	1.4(0.2‐4)	.01	1(1‐1.2)	.02	1(0.9‐1)	.07
Tp‐e	2(0.9‐4)	<.0001	3(1‐15)	.01	0.4(0.2‐0.9)	.02	0.5(0.2‐1)	.09
iCEB	0.2(−6‐11)	.23	0.3(−22‐4)	.45	2(0.1‐43)	.41	2(0.1‐5)	.42
TDR	4(1‐5)	<.0001	3(0.9‐14)	.01	1(1‐3)	.03	0.4(0.2‐1)	.09

Abbreviations: iCEB, index of cardiac electrophysiological balance; ICU, intensive care unit; OR (CI), Odds ratio (confidence interval); TDR, transmural dispersion of repolarization; Tp‐e, T from peak to end interval.

### 3.2 Multivariate analysis

Baseline clinical characteristics and ECG markers that showed significant association in univariate analysis were selected for final multivariate analysis. Increased QTc interval, Tp‐e interval, and TDR were independent predictors of increased length of ICU stay (*P* < .0001). Increasing age (*P* < .0001) followed by increased QTc interval (*P* = .02) and history of chronic lung disease (*P* = .04) were independent predictors of extensive degree of lung damage as assessed by CT score. The independent predictors of in‐hospital cardiac arrest were increased QTc interval (*P* = .02) followed by increasing age (*P* = .04) and increased Tp‐e interval (*P* = .04) (Table 3).

**TABLE 3:** Multivariate analysis of baseline ECG markers and clinical characteristics with clinical outcomes^a^, ^b^

Variables	ICU length stay		Lung injury		Cardiac arrest	
	OR(CI)	*P*	OR(CI)	*P*	OR(CI)	*P*
QTc	0.3(0.2‐1.3)	<.0001	1.5(0.1‐3)	.02	1.2(1‐1.4)	.02
Tp‐e	4(2‐7)	<.0001	3(−17‐0.4)	.06	0.3(0.1‐0.9)	.04
TDR	2(3‐8)	<.0001	1.5(−4‐15)	.06	0.5(0.1‐1)	.05
Old age	0.1(−0.0‐0.3)	.23	0.5(0.5‐1.3)	<.0001	1(1‐1.1)	.04
Chronic lung disease	0.0(−6‐3)	.59	0.2(0.1‐30)	.04	0.3(0.0‐3)	.36
Male	0.0(−5‐3)	.63	0.1(−20‐8)	.34	0.9(0.1‐4)	.92

Abbreviations: ICU, intensive care unit; OR (CI), Odds ratio (confidence interval); TDR, transmural dispersion of repolarization; Tp‐e, T from peak to end interval.

^a^ Baseline clinical characteristics, including age, sex, hypertension, diabetes mellitus, chronic lung disease, smoking, body mass index, and previous coronary artery disease and ECG markers with *P* value < .05 in the univariate logistic regression model, were entered as predictors of adverse clinical outcome in the final multivariate regression model.

^b^ Only variables significant with *P* value < .05 are displayed in the table.

## 4 DISCUSSION

Given the high rates of acute phase morbidity and mortality associated with rapidly spreading COVID‐19 pneumonia, there is an urgent need to identify prognostic markers that can help the physician in the rapid triage of patients and optimized allocations of health‐care resources.^11^ In the literature, there is scant data as of yet that address the predictors of increased risk of adverse outcome of COVID‐19 pneumonia, particularly early in patients’ clinical course.^12^

In the present study, patients with increased baseline QTc were at increased risk for prolonged ICU stay, cardiac arrest, and extensive lung injury. Also, patients with increased Tp‐e at presentation were at increased risk of prolonged ICU stay and cardiac arrest, while patients with increased TDR were at increased risk for prolonged ICU stay.

Previous clinical studies on COVID‐19 infection assessed a range of different disease outcomes, including hospitalization, mechanical ventilation, and in‐hospital fatality rate with different results.^13^, ^14^ Among the United State population, short‐term case fatality rates are estimated to be between 1.8% and 3.4%, which is higher than the 1.4% estimate from China. Nevertheless, short‐term case fatality rates in case series of hospitalized patients and patients with severe pneumonia have been much higher, ranging from 10.2% to 67%.^13^, ^15^

The prevalence of ICU admission and requiring mechanical ventilation was controversial among prior studies.^16^, ^17^ Nowak B et al reported that ICU admission in 16% and 15.4% of enrolled patients required mechanical ventilation.^16^ On the other hand, a recent study from New York City reported that 33% of patients with COVID‐19 infection required mechanical ventilation.^17^ These differences in ICU admission rate and use of mechanical ventilation could be attributed to the heterogeneity of the disease severity illness and a limited number of available ventilators or ICU beds.

Cardiac arrhythmia and arrest are common cardiac manifestations in COVID‐19 patients, particularly among ICU patients compared to the non‐ICU patients (44.4% vs 6.9%).^18^, ^19^ It is notable that cardiac injury, as a common complication (19.7%) in the setting of COVID‐19 infection, is associated with an unexpectedly high risk of mortality during hospitalization.^20^ Cardiac injury caused by myocarditis secondary to COVID‐19 infection, causing electrical disturbances leading to arrhythmias or even sudden death, could be a potential explanation for cardiac arrest occurred in the setting of COVID‐19 infection even in the absence of a history of cardiovascular diseases.^21^ Cardiac arrest or injury in patients with COVID‐19 could be attributed to myocardial stress caused by metabolic and electrolyte abnormalities, hypoxia, increased endogenous catecholamine release, or inflammation of myocardial cells, whether the patient has preexisting coronary artery disease or not.^18^, ^19^

Regarding ECG markers of repolarization disturbances, QTc, Tp‐e interval, and TDR have a prognostic significance in the prediction of increased risk of malignant arrhythmia and morality in patients with certain cardiovascular diseases, such as coronary artery disease and myocarditis.^22^ ECG changes, such as QTc prolongation, conduction and repolarization abnormalities, and ventricular arrhythmias, may reflect myocardial injury directly or indirectly associated with COVID‐19 pneumonia.^23^ Abrams MP et al found that patients who died of cardiac arrest were more likely to have a longer QTc on admission, receiving mechanical ventilation, preexisting chronic lung disease, and development of ventricular ectopy.^24^ Along the same line, the prognostic value of ECG disturbances at presentation to the emergency department was assessed in a study conducted by Elias P et al who enrolled 1258 adults with COVID‐19. They found a significant increase in the event rate of death and respiratory failure when abnormal ECG changes, such as atrial arrhythmia, right ventricular strain, and ST‐segment abnormalities, were incorporated into multivariable regression, with higher prognostic value than every other variable in the model except for abnormal pulmonary vital signs.^25^

The present study has several limitations. This is a retrospective study, and the data of patients were extracted from medical patient history or records, so findings may be not generalizable. The majority of enrolled patients (85%) were found to be positive PCR for SARS‐CoV‐2 in the present study, while the reaming patients (15%) with negative PCR test had probable or strongly suspected COVID‐19 pneumonia based on clinical features and history, radiological findings, and clear epidemiological history. Currently, it has been found that the PCR testing for COVID‐19 has limited sensitivity, whereas lung CT may reveal early pulmonary alterations consistent with COVID‐19 infection in patients with an initial negative PCR exam.^26^ We did not assess the possibility of myocarditis because of the absence of data on serum markers and echocardiographic examination for most of our enrolled patients because of logistical limitations. The specific rhythm type that occurred in those who died of COVID‐19 was not reported in medical records because of a lack of telemetry information or serial ECGs. Therefore, we cannot verify the specific type of cardiac rhythm or arrhythmic events that occurred during ICU stay or prior to death. Data from larger populations are required to further confirm the role of ECG markers as a clinical predictor in the long‐term follow‐up studies.

## 5 CONCLUSION

COVID‐19 pneumonia patients with baseline ECG repolarization changes, particularly increased QTc interval, were at increased risk for in‐hospital cardiac arrest, prolonged length of ICU stay, and extensive lung injury. Repolarization abnormalities on baseline ECG may be useful prognostic markers in patients with COVID‐19 pneumonia.

## CONFLICT OF INTEREST

The authors declare that they have no conflict of interest.
